# SLFN11 expression correlates with immune microenvironment and predicts prognosis in melanoma

**DOI:** 10.3389/fimmu.2025.1607056

**Published:** 2025-09-22

**Authors:** Huancheng Zeng, Guishan Chen, Yutong Fang, Jundong Wu, Qiongzhi Jiang, Rendong Zhang

**Affiliations:** ^1^ Department of Breast Surgery, Cancer Hospital of Shantou University Medical College, Shantou, Guangdong, China; ^2^ Endocrinology and Metabolism Department, The First Affiliated Hospital of Shantou University Medical College, Shantou, Guangdong, China; ^3^ Department of Radiation Oncology, Cancer Hospital of Shantou University Medical College, Shantou, Guangdong, China

**Keywords:** SLFN11, melanoma, immunotherapy, therapeutic target, biomarker

## Abstract

**Background:**

Schlafen family member 11 (SLFN11) has been implicated in cancer biology and immune modulation, but its expression patterns, prognostic value, and role in tumor immunity in melanoma remain incompletely defined.

**Methods:**

Through multi-omics analyses of public databases (The Human Protein Atlas, TIMER2, BEST) and functional validation, we characterized SLFN11 in melanoma. Functional assays were conducted in SLFN11-overexpressing melanoma cells to evaluate effects on M0 macrophage polarization, recruitment of macrophages and CD8⁺ T cells, and CD8⁺ T cell cytotoxic activity.

**Results:**

SLFN11 mRNA levels are reduced in skin cutaneous melanoma (SKCM) compared to normal skin, yet higher in metastatic lesions than in primary tumors. High SLFN11 expression correlates with favorable overall and progression-free survival across multiple independent melanoma cohorts, with consistent prognostic value across clinical subgroups (tumor stages, nodal/metastatic status). Multivariable Cox regression analysis, adjusting for factors like gender, age, and pathologic T/N/M stages, confirmed SLFN11 expression as an independent predictor of favorable overall survival. SLFN11 expression associates with enhanced infiltration of immune cells along with co-expression of immune checkpoint molecules. Furthermore, SLFN11 expression is associated with favorable prognosis in immunotherapy-treated patients. Functional assays show that SLFN11-overexpressing melanoma cells promote M0 macrophage polarization toward an M1 phenotype, enhance recruitment of macrophages and CD8⁺ T cells, and slightly increase CD8⁺ T cell cytotoxic activity.

**Conclusions:**

These findings provide evidence that SLFN11 is associated with immune microenvironment changes in melanoma, correlates with favorable prognosis, and may be linked to immunotherapy response, supporting its potential as a candidate biomarker and therapeutic target for further investigation.

## 1 Introduction

Melanoma, a highly aggressive skin cancer, has benefited significantly from immune checkpoint blockade (ICB) therapies. Yet, primary and acquired resistance remain major obstacles, with only a subset of patients achieving durable responses (1, 2). Current predictive biomarkers such as PD-L1 expression and tumor mutational burden (TMB) have limited utility (3, 4) highlighting the need for novel targets and biomarkers to refine patient stratification and improve therapeutic outcomes (5–7).

The Schlafen (SLFN) family, involved in cell proliferation and immune regulation, includes SLFN11—a DNA/RNA helicase with well-documented roles in sensitizing tumors to DNA-damaging therapies (e.g., PARP inhibitors, platinum agents) in small cell lung cancer and ovarian cancer (8) (9). Emerging evidence suggests context-dependent immune regulatory functions: in hepatocellular carcinoma, SLFN11 suppresses M2 macrophage polarization to enhance anti-PD-1 efficacy (10), while in breast cancer, its epigenetic upregulation synergizes with targeted therapies (11). However, SLFN11’s expression patterns, prognostic significance, and immune-related roles in melanoma, particularly its association with ICB response, remain largely uncharacterized.

Here, we present a comprehensive analysis of SLFN11 in melanoma, integrating multi-omics data from public databases with *in vitro* functional validation. We aimed to: first, characterize SLFN11 expression across melanoma stages (including primary versus metastatic) and compare it with normal tissues and other cancers; second, evaluate its prognostic value for survival outcomes across diverse melanoma cohorts; third, explore correlations with immune cell infiltration, checkpoint molecules, and antigen presentation genes; and fourth, validate its potential immunomodulatory functions (macrophage polarization and T cell recruitment and activity) *in vitro*. These analyses may offer insights into SLFN11’s role in melanoma biology and suggest its potential as a candidate predictive biomarker for immunotherapy response, with preliminary implications for future therapeutic strategy development.

## 2 Methods

### 2.1 Expression profiling of SLFN11

To compare SLFN11 mRNA levels between skin cutaneous melanoma (SKCM) and normal skin, we utilized the TNMplot database (https://tnmplot.com/analysis/), which provides normalized RNA-seq data for tumor and adjacent normal tissues (12). Within the TCGA-SKCM cohort, we extracted RNA-seq data, preprocessed and curated in the Broad Institute’s BEST database (which integrates resources from the Cancer Cell Line Encyclopedia and Tumor Gene Expression Analysis), to compare SLFN11 expression between primary melanoma tumors and metastatic lesions, as well as across clinical stages (T stages, nodal [N] status, and metastatic [M] status). Additionally, we analyzed SLFN11 mRNA levels in melanoma samples with and without ulceration using TCGA-SKCM data to explore associations with this histopathological feature (13). For assessments of SLFN11 expression in the context of temozolomide treatment, we retrieved data from the GSE19293 dataset, which includes gene expression profiles of melanoma samples from patients treated with temozolomide (13, 14).The comparison of SLFN11 mRNA expression between primary and metastatic melanoma tissues was further analyzed using TIMER 2.0 (http://timer.cistrome.org/) (15, 16).

### 2.2 Survival and prognostic analyses of SLFN11 in melanoma

We analyzed survival outcome data from multiple independent cohorts, including TCGA-SKCM and GEO datasets (GSE19234, GSE54467, GSE99898, GSE190113, GSE22154, GSE53118, GSE65904, GSE133713), using the Broad Institute’s BEST database (which integrates the Cancer Cell Line Encyclopedia and Tumor Gene Expression Analysis). Patients in each cohort were stratified into high and low SLFN11 expression groups using the median expression as the cutoff. Kaplan-Meier survival analyses were performed to assess differences in overall survival (OS) and progression-free survival (PFS), with statistical significance determined by the log-rank test. Subgroup survival analyses within the TCGA-SKCM cohort were further conducted by stratifying patients according to tumor stages (T1, T2, T3), metastatic statuses (M0, M1), and nodal stages (N1, N2, N3, N4) to examine the consistency of SLFN11’s prognostic value across different clinical subgroups. Additionally, multivariable Cox proportional hazards regression analysis was performed in the TCGA-SKCM cohort, adjusting for clinical factors including gender, age, and pathologic T, N, and M stages, to determine whether SLFN11 expression independently predicts OS. Hazard ratios (HR) and 95% confidence intervals (CI) were calculated to quantify the association.

### 2.3 Tumor immune microenvironment characterization

For immune cell infiltration analysis, we utilized the TIMER2 database, which employs deconvolution algorithms to estimate immune cell fractions from bulk RNA-seq data. We calculated Spearman’s rank correlation coefficients to assess the relationship between SLFN11 mRNA expression and the infiltration levels of specific immune cell populations, including pro-inflammatory subsets (CD8^+^ T cells, M1 macrophages, natural killer cells) and immunosuppressive regulators [regulatory T cells, cancer-associated fibroblasts (CAFs)] and other immunosuppressive populations (M2 macrophages, myeloid-derived suppressor cells [MDSCs]).

We analyzed RNA-seq data from the TCGA-SKCM cohort, focusing on key immune checkpoint genes (BTLA, CD274, PDCD1LG2, CTLA-4, TIGIT, HAVCR2, LAG-3, PDCD1 [PD-1]), and computed Spearman’s correlation coefficients to quantify these associations. We extended these analyses to multiple independent melanoma datasets from the Broad Institute’s BEST database, including GSE100797, GSE78220, GSE190113, GSE53894, GSE133713, GSE22153, GSE65904, GSE35447, GSE1118, GSE19234, GSE2154, GSE21923, and GSE99898. For each dataset, we calculated Spearman’s correlations between SLFN11 expression and: (1) immune infiltration scores (CD8^+^ T cells, macrophages, dendritic cells, neutrophils, B cells); (2) expression of immunoinhibitor molecules; (3) expression of human leukocyte antigen (HLA) family genes; and (4) expression of antigen processing transporters.

### 2.4 Gene set enrichment and molecular pathway analysis

Gene Set Enrichment Analysis (GSEA) was conducted using the Broad Institute’s BEST database (Broad Institute’s Cancer Cell Line Encyclopedia and Tumor Gene Expression Analysis). Hallmark gene sets from the Molecular Signatures Database (MSigDB) were interrogated to identify pathways associated with SLFN11-high melanomas. Normalized enrichment scores (NES) and false discovery rates (FDR) were calculated using 1,000 permutations, with significance defined as FDR <0.25 and p < 0.05.

### 2.5 Analysis of SLFN11 expression and prognostic associations in immunotherapy-treated patients

We analyzed clinical and gene expression data from publicly available cohorts of patients treated with immune checkpoint inhibitors (ICIs) or cellular therapies. Immunotherapy cohort data were retrieved from the Broad Institute’s BEST database, including cohorts of patients treated with anti-CTLA-4 (ipilimumab), anti-PD-1 (pembrolizumab, nivolumab), anti-PD-L1 (atezolizumab, durvalumab), and CAR-T cell therapy. Patients in each cohort were stratified into high and low SLFN11 expression groups using the median expression level of SLFN11 as the cutoff, consistent with the stratification method used for survival analyses in untreated cohorts. Kaplan-Meier survival curves were generated to compare overall survival (OS) and progression-free survival (PFS) between high and low SLFN11 expression groups, with statistical significance for differences in survival outcomes determined using the log-rank test, and hazard ratios (HR) and 95% confidence intervals (CI) calculated to quantify the strength of association between SLFN11 expression and survival.

### 2.6 Cell culture and stable cell line generation

HEK-293T cells (American Type Culture Collection, ATCC) and A375 melanoma cells (purchased from Procell Life Science & Technology Co., Ltd.) were cultured in DMEM medium (Thermo Fisher Scientific) supplemented with 10% fetal bovine serum (FBS) and 1% penicillin/streptomycin. SK-Mel-246 melanoma cells and THP-1 cells (both from ATCC) were maintained in RPMI-1640 medium containing 10% FBS and 1% penicillin/streptomycin. SK-Mel-246 was selected primarily for its well-documented utility in studies investigating T cell-mediated cytotoxicity and tumor-immune interactions (5), where it has been successfully employed in CD8^+^ T cell co-culture assays to evaluate antitumor responses. A375, originating from a female patient, was included to complement the male-derived SK-Mel-246, expanding the gender diversity of our experimental models.

To generate stable SLFN11-overexpressing (SLFN11-OE) and negative control (NC) cell lines, cells were transfected with pcDNA3.1-SLFN11 or empty vector (pcDNA3.1) using Lipofectamine 3000 (Invitrogen), following the manufacturer’s instruction. Puromycin (Solarbio) was used to select stably transfected cell lines. Mycoplasma testing was routinely performed using the MycoBlue Mycoplasma Detector (Vazyme).

### 2.7 qPCR analysis

Total RNA from co-cultured macrophages was extracted using TRIzol (Invitrogen). qPCR was conducted with the ES Science 2X Super SYBR Green qRT-PCR Master Mix. Real-time RT-PCR analyses were performed using a CFX96 Touch Real-Time PCR Detection System. The qRT-PCR primers used to determine target gene expression levels are shown in **Supplementary Table**. Relative mRNA expression was calculated using the 2−ΔΔCt method normalized to GAPDH.

### 2.8 Western blotting

Cell lysates in the WB buffer were denatured at 95 °C for 10 min, followed by electrophoresis and transfer onto nitrocellulose membrane. The membrane was blocked with 5% fat-free milk, incubated with primary antibodies (SLFN11, CST) at 4 °C overnight, and labeled with horseradish peroxidase (HRP)-conjugated secondary antibodies (CST). Fluorescence intensities of the bands were normalized to GAPDH (CST). Blots were visualized by using BeyoECL Moon (Beyotime).

### 2.9 Macrophage polarization assay

For M0 macrophage polarization, THP-1 cells were seeded in 6-well plates at a density of 5 × 10^5^ cells/well and treated with 100 nM phorbol 12-myristate 13-acetate (PMA; Sigma-Aldrich) for 48 hours. To validate polarization efficiency, M0 macrophages were further stimulated to polarize into M1 or M2 subsets: M1 polarization was induced by treating M0 macrophages with 100 ng/mL lipopolysaccharide (LPS; Sigma-Aldrich) + 20 ng/mL recombinant human interferon-γ (IFNγ; PeproTech) for 48 hours, while M2 polarization was induced with 20 ng/mL recombinant human interleukin-4 (IL4; PeproTech) + 20 ng/mL recombinant human interleukin-13 (IL13; PeproTech) for 48 hours. Polarization was confirmed by qPCR analysis of M1 (NOS2, CXCL10, TNFα) and M2 (CD163, ARG1, CD206) marker genes, with untreated THP-1 monocytes and PMA-induced M0 macrophages included as controls to verify the unpolarized phenotype of M0 cells.

### 2.10 Co-culture of M0 macrophage with melanoma cells

For co-culture, SLFN11-OE or NC melanoma cells (1×10^5^ cells/well) were seeded in the lower chamber of 12-well plates, while PMA-induced M0 macrophages (1×10^5^ cells/well) were plated in the upper chamber of 0.4 μm pore-size Transwell inserts (Corning). Cells were co-cultured for 48 hours in RPMI-1640 medium supplemented with 10% FBS, allowing bidirectional cytokine communication without direct cell-cell contact. After co-culture, macrophages in the Transwell upper chambers were carefully collected by gentle scraping for qPCR.

### 2.11 ELISA

After co-culture, macrophages were isolated and cultured in fresh medium for 48 hours. Supernatants were collected, and CXCL10 secretion was quantified using a Human CXCL10 ELISA Kit (Proteintech) following the manufacturer’s protocol. Absorbance was measured at 450 nm with a microplate reader (BioTek).

### 2.12 Transwell migration assays for tumor cell-mediated chemotaxis of macrophages and CD8^+^ T cells

THP-1-derived M0 macrophages and primary human CD8^+^ T cells were labeled with 5 μM CFSE (Invitrogen) in serum-free RPMI 1640 medium for 15 minutes at 37°C, followed by two washes with complete medium to remove unincorporated dye. For macrophage migration, CFSE-labeled macrophages (1 × 10^5^ cells/well) were seeded into the upper chamber of 24-well transwell plates (8 μm, Corning), with the lower chamber containing 800 μL complete medium and SLFN11-overexpressing (SLFN11-OE) or negative control (NC) tumor cells (SK-Mel-246 or A375) at 2 × 10^5^ cells/well. After 48 hours of incubation, non-migrated cells in the upper chamber were removed with a cotton swab; migrated cells on the lower membrane surface were fixed with 4% paraformaldehyde, stained with 0.1% crystal violet (Sigma-Aldrich), imaged, and quantified by counting in three random fields, while migrated cells in the lower chamber were analyzed by flow cytometry to determine the number of CFSE^+^ macrophages. For CD8^+^ T cell migration, CFSE-labeled CD8^+^ T cell (1 × 10^5^ cells/well) were seeded into the upper chamber of 24-well transwell plates (5 μm, Corning), with the lower chamber containing SLFN11-OE or NC tumor cells under the same conditions; after 48 hours, migrated CD8^+^ T cells in the lower chamber were collected and analyzed by flow cytometry to quantify CFSE^+^ cells.

### 2.13 Co-culture of CD8^+^ T cells with melanoma cells

Primary human CD8^+^ T cells were isolated from peripheral blood mononuclear cells (PBMCs) of healthy donors using the EasySep Human CD8^+^ T Cell Isolation Kit (Stemcell Technologies). Isolated CD8^+^ T cells were resuspended in RPMI 1640 medium (Gibco) supplemented with 10% heat-inactivated fetal bovine serum (FBS; Gibco), 100 U/mL penicillin, 100 μg/mL streptomycin, and 2 mM L-glutamine (Thermo Fisher Scientific), then activated with human CD3/CD28 (Stemcell Technologies) for 72 hours. CD8^+^ T cells were maintained in the medium supplemented with 20 ng/mL recombinant human IL-2 (PeproTech) until use in co-culture assays.

For co-culture assays, SLFN11-OE or NC melanoma cells were seeded in 6-well plates at a density of 1 × 10^5^ cells/well and allowed to adhere overnight. Activated CD8^+^ T cells were added to each well at a 5:1 effector-to-target ratio (5 × 10^5^ T cells per well) and co-cultured for 72 hours. After co-culture, CD8+ T cells were harvested, washed with PBS, and stained for flow cytometric analysis.

### 2.14 Flow cytometry

Flow cytometric analysis of migrated cells in transwell assays involved collecting samples from the lower chamber (containing migrated macrophages or CD8^+^ T cells) and analyzing them via flow cytometry. For CFSE-labeled macrophages or CD8^+^ T cells, the CFSE^+^ population was gated to quantify migrated cells. Absolute counts of CFSE^+^ cells across groups were compared to determine the chemoattractive capacity of SLFN11-OE versus NC tumor cells.

For flow cytometric analysis of intracellular cytokines in co-cultured CD8^+^ T cells, cells were stimulated with Cell Stimulation Cocktail plus protein transport inhibitors (eBioscience) for 4 hours at 37°C with 5% CO_2_. After stimulation, cells were centrifuged and washed with PBS, then stained with a viability dye (BioLegend) for 15 minutes at room temperature in the dark to exclude dead cells. Following viability staining, cells were incubated with fluorochrome-conjugated antibodies against CD3 (Clone SK7, Biolegend) and CD8 (Clone SK7, BD BioLegend) for 30 minutes at 4°C in the dark, washed with PBS, and then fixed and permeabilized using Fixation Buffer (eBioscience). Intracellular staining was performed with a fluorochrome-conjugated antibody against IFN-γ (Clone 4S.B3, BioLegend) for 30 minutes at 4°C in the dark, followed by washing with permeabilization buffer. Data acquisition was performed on the Cytek Aurora, and the percentages of cells were calculated using the FlowJo software.

### 2.15 Statistical analysis

Kaplan - Meier curves with log - rank tests compared overall survival (OS)/progression - free survival (PFS) between SLFN11 - high and SLFN11 - low subgroups. Spearman’s rank correlation assessed relationships between SLFN expression, immune checkpoint genes and immune cell scores (P < 0.05). Cox regression analyses were performed using the survival package in R to calculate the hazard ratios (HR). Flow cytometry and qPCR, Elisa data were analyzed in GraphPad Prism, and statistical significance was determined by unpaired Student’s t-test, with a p-value < 0.05 considered statistically significant.

## 3 Results

### 3.1 Expression patterns of SLFN11 in melanoma

**Figure 1:**
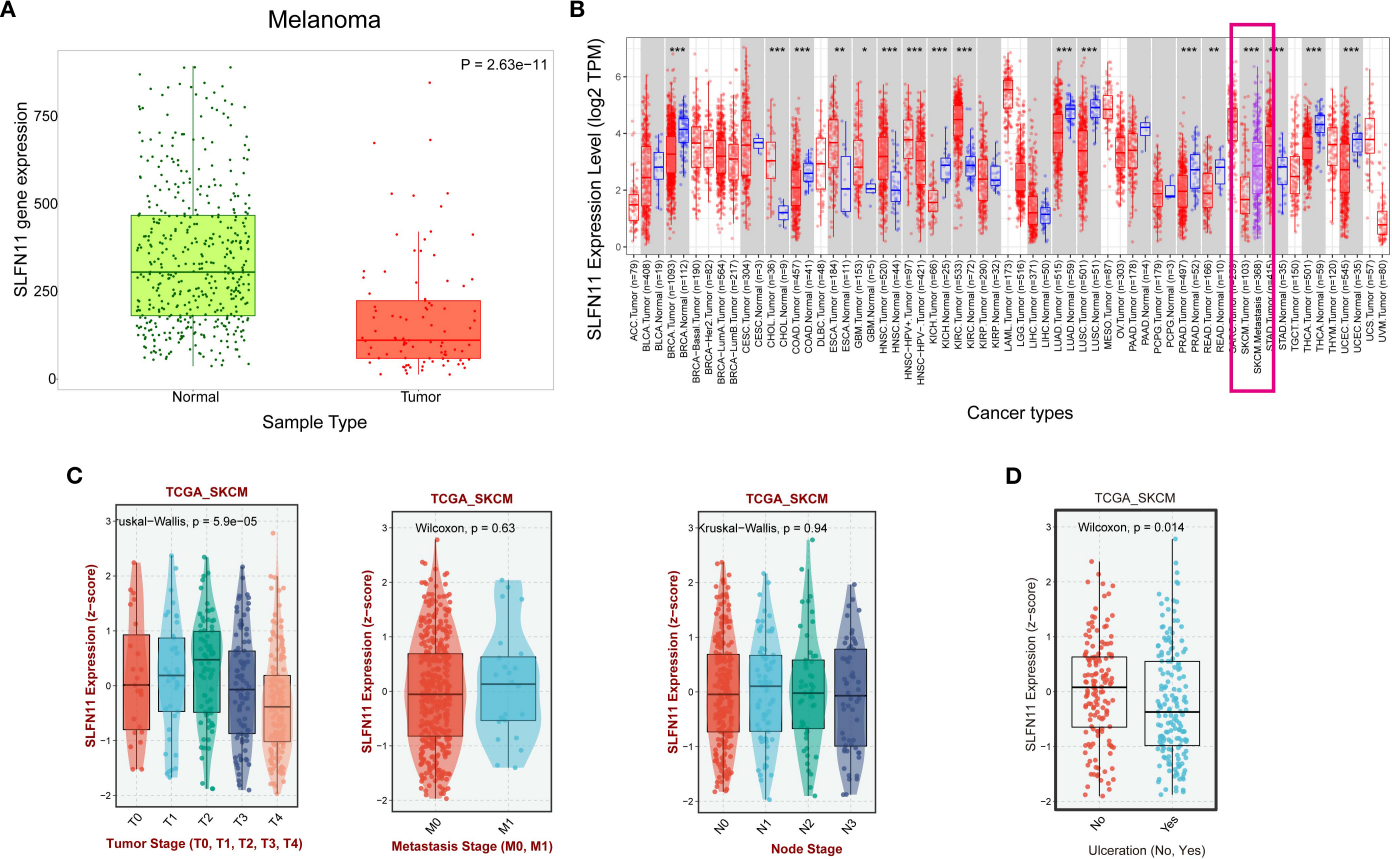
SLFN11 expression patterns in melanoma and its association with clinical pathological features. **(A)** Comparison of SLFN11 expression levels (log2 TPM) in melanoma and normal tissue. **(B)** SLFN11 expression levels in primary melanoma versus metastatic melanoma from the TIMER2 database **(C)** SLFN11 expression in melanoma across different T stages, nodal (N) status, and metastatic (M) status within the TCGA_SKCM cohort. **(D)** SLFN11 expression (z−score) in melanoma with and without ulceration within the TCGA_SKCM cohort. The Wilcoxon test was used for statistical analysis.

To characterize the expression patterns of SLFN11 in melanoma, we first compared the mRNA levels between skin cutaneous melanoma (SKCM) tissues and normal skin using the TNMplot database (https://tnmplot.com/analysis/). This analysis revealed a significant downregulation of SLFN11 in SKCM tumor tissues compared to normal skin (P < 0.001) (**Figure 1A**). Within the TCGA - SKCM cohort, metastatic lesions exhibited higher SLFN11 expression than primary tumors (P < 0.001) (**Figure 1B**). Further analysis across clinical stages showed a downregulation of SLFN11 in T4 - stage melanomas compared to T1 - T3 stages. No significant trends were observed across nodal (N) or metastatic (M) stages (**Figure 1C**). Meanwhile, SLFN11 expression was significantly reduced in melanomas with ulceration relative to non - ulcerated tumors, suggesting an association with this aggressive histopathological feature (**Figure 1D**). Collectively, these expression patterns, including lower SLFN11 in primary SKCM vs. normal skin, higher levels in metastases vs. primaries, and reduced expression in aggressive subtypes (T4 stage, ulcerated tumors), hint that SLFN11 may be linked to melanoma progression and biological aggressiveness. Given this potential association with tumor behavior, we next investigated whether SLFN11 expression correlates with survival outcomes to clarify its prognostic relevance in melanoma.

### 3.2 SLFN11 expression correlates with favorable survival outcomes in melanoma across multiple independent melanoma cohorts and clinical subgroups

**Figure 2:**
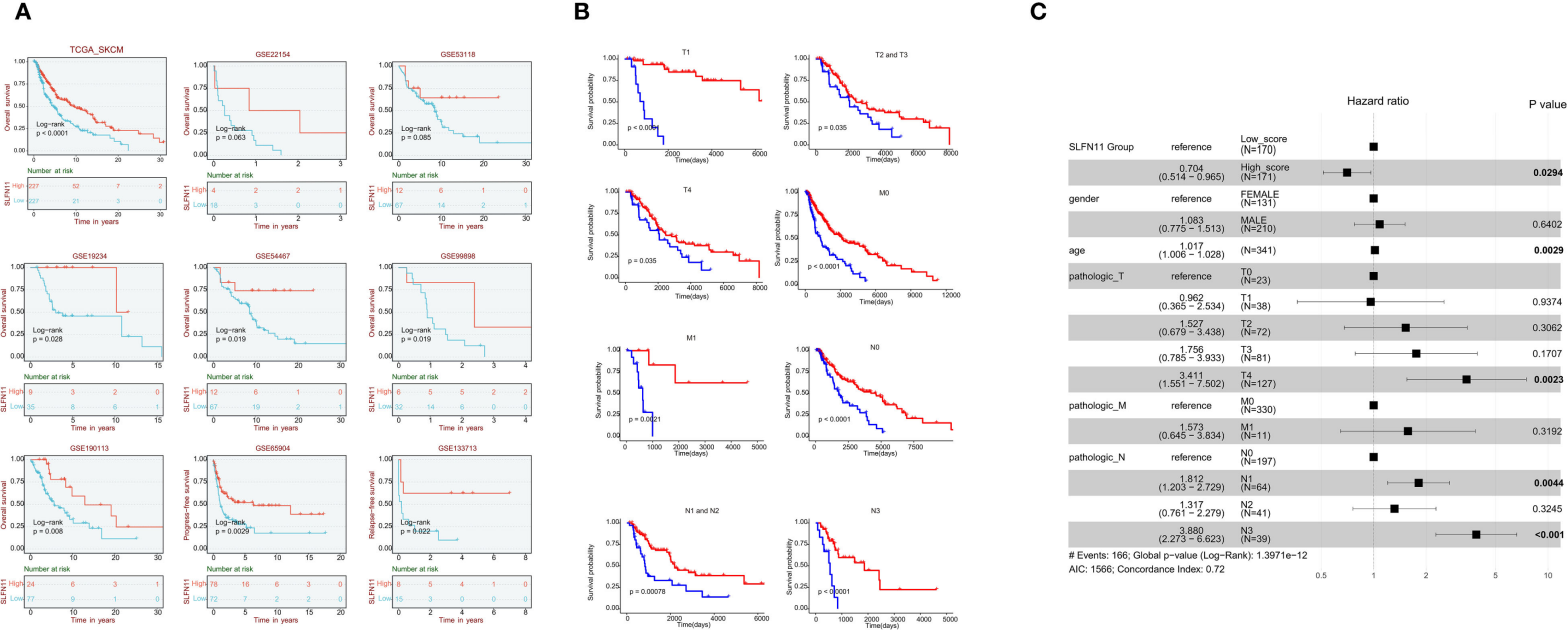
Survival outcomes associated with SLFN11 expression in melanoma **(A)** Survival curves showing overall survival (OS) in five independent melanoma cohorts (TCGA, GSE19234, GSE54467, GSE99898, GSE190113) and progression-free survival (PFS) in two cohorts (GSE65904, GSE133713) stratified by SLFN11 expression levels. Significance levels are as follows: TCGA (p < 0.001), GSE19234 (p = 0.028), GSE54467 (p = 0.019), GSE99898 (p = 0.019), GSE190113 (p = 0.008), GSE65904 (p = 0.0029), GSE133713 (p = 0.022); borderline significance is noted for GSE22154 (p = 0.063) and GSE53118 (p = 0.085). **(B)** Subgroup survival analyses of overall survival (OS) in the TCGA-SKCM cohort, stratified by SLFN11 expression across different clinical subgroups: T1, T2, and T3 tumor stages; M0 and M1 metastatic status; and N1, N2, N3, and N4 nodal stages, demonstrating the consistent favorable prognostic value of high SLFN11 expression across various disease stages. **(C)**. Forest plot of multivariable Cox regression analysis in the TCGA-SKCM cohort, adjusting for clinical factors including age, gender, and pathologic T/N/M stages. Variables with a p-value < 0.05 are highlighted in bold. The Akaike Information Criterion (AIC = 1566) quantifies the relative quality of this statistical model, balancing goodness - of - fit and model complexity. A lower AIC generally indicates a better - fitting model relative to others; here, it helps assess how well this multivariable Cox model explains the OS data while accounting for included clinical predictors. Additionally, the Concordance Index (0.72) reflects the model’s ability to differentiate between patients with different survival outcomes, with values closer to 1 signifying stronger predictive performance.

Survival analysis of multiple independent cohorts yielded key findings: Patients with high SLFN11 expression exhibited significantly prolonged overall survival (OS) in five independent cohorts (TCGA, p<0.001; GSE19234, p=0.028; GSE54467,

p=0.019; GSE99898, p=0.019; GSE190113, p=0.008). Borderline significance was observed in GSE22154 (p=0.063) and GSE53118 (p=0.085). Progression - free survival (PFS) benefits were consistently observed in both GSE65904 (p=0.0029) and GSE133713 (p=0.022) (**Figure 2A**).

Further subgroup survival analyses within the TCGA - SKCM cohort revealed that high SLFN11 expression was associated with significantly longer OS across different clinical subgroups, including T1, T2, and T3 tumor stages; M0 and M1 metastatic statuses; and N1, N2, N3, and N4 nodal stages. These subgroup analyses reinforce that the favorable prognostic value of SLFN11 is consistent across different disease stages in melanoma (**Figure 2B**).

Multivariable Cox regression analysis in the TCGA - SKCM cohort adjusted for clinical factors including gender, age, and pathologic T, N, M stages. high SLFN11 expression was an independent predictor of favorable OS (HR = 0.704, 95% CI: 0.514–0.965, p = 0.0294), with other factors like age, pathologic T4 stage, and certain nodal stages also having prognostic significance (**Figure 2C**). Since SLFN11 expression independently predicts favorable survival in melanoma, we hypothesized this prognostic value could be associated with changes in the tumor immune microenvironment, a well-documented determinant of melanoma progression and patient outcomes (17, 18). We therefore next explored the correlation between SLFN11 expression and immune cell infiltration, immune checkpoint molecule expression, and antigen presentation gene levels to understand how the tumor immune microenvironment may relate to SLFN11’s prognostic role in melanoma.

### 3.3 Correlation of SLFN11 with immune infiltration cell scores, checkpoints gene expression, and antigen presentation genes in melanoma

**Figure 3:**
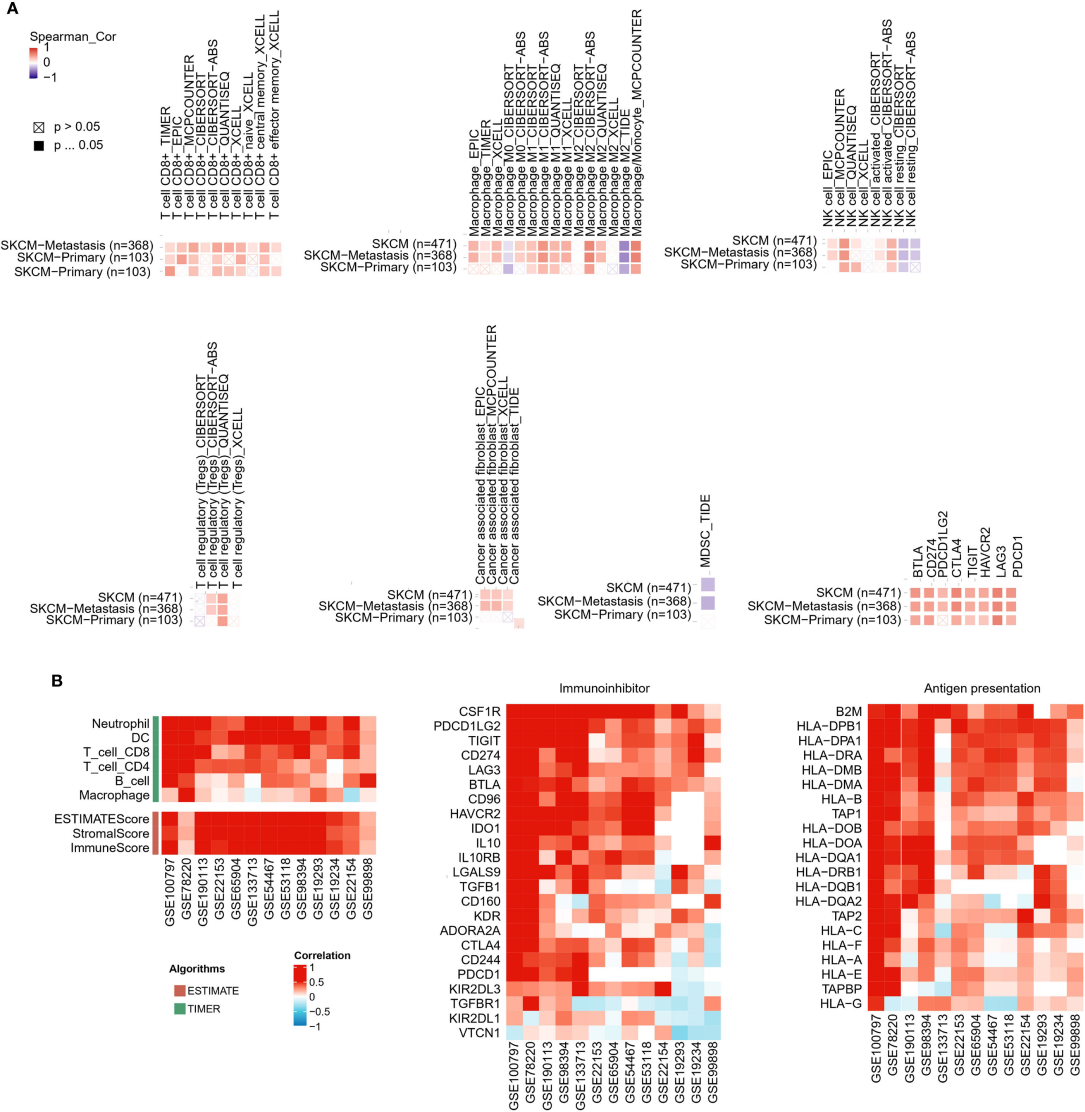
Correlation of SLFN11 with immune infiltration, immune checkpoints, and antigen presentation in melanoma. **(A)** Heatmap of Spearman’s correlation between SLFN11 RNA levels and immune cell infiltration scores and immune checkpoint genes across melanoma. Red: positive association; blue: negative. **(B)** Consistency of SLFN11 correlations across multiple melanoma datasets (GSE100797, GSE78220, GSE190113, GSE53894, GSE133713, GSE22153, GSE65904, GSE35447, GSE1118, GSE19234, GSE2154, GSE21923, GSE99898). Displayed are correlations of SLFN11 with genes related to pro-inflammatory immune infiltration markers (T cells, macrophages, dendritic cells [DC], neutrophils, B cells), immunoinhibitor molecules, human leukocyte antigen (HLA) genes, and antigen processing transporters.

Immunoinfiltration analysis using the TIMER2 database revealed significant correlations between SLFN11 expression and the immune landscape. In melanoma, elevated SLFN11 expression positively correlated with the infiltration of pro-inflammatory immune cells (CD8^+^ T cells, M1 macrophages, natural killer cells) and immunosuppressive regulators (regulatory T cells Tregs, cancer-associated fibroblasts [CAFs]). In contrast, it showed negative associations with immunosuppressive populations (M2 macrophages, myeloid-derived suppressor cells [MDSCs]) (**Figure 3A**). SLFN11 demonstrated co-expression patterns with immune checkpoint molecules in melanoma, including BTLA, PD-L1 (CD274), PD-L2 (PDCD1LG2), CTLA-4, TIGIT, TIM-3 (HAVCR2), LAG-3, and PD-1 (PDCD1), suggesting its potential role in modulating immune checkpoint-mediated tumor evasion (**Figure 3A**).

We further extended this analysis to multiple melanoma datasets (GSE100797, GSE78220, GSE190113, GSE53894, GSE133713, GSE22153, GSE65904, GSE35447, GSE1118, GSE19234, GSE2154, GSE21923, GSE99898). Across these datasets, consistent trends emerged: SLFN11 expression positively correlated with markers of pro-inflammatory immune infiltration (CD8^+^ T cells, macrophages, dendritic cells, neutrophils, B cells) and immunoinhibitor molecules (**Figure 3B**). In parallel, across the GSE melanoma datasets, SLFN11 expression positively correlated with multiple human leukocyte antigen (HLA) genes (**Figure 3B**). In general, these results demonstrate that high SLFN11 expression in melanoma is associated with a pro-inflammatory tumor immune microenvironment characterized by increased infiltration of cytotoxic immune cells and co-expression of key immune checkpoint molecules. This observation raises the possibility that SLFN11 expression may be linked to a tumor immune microenvironment that is more favorable for immunotherapy responses, suggesting its potential association with prognosis in patients receiving immune checkpoint inhibitors.

We therefore next analyzed SLFN11’s prognostic value in immunotherapy-treated cohorts.

### 3.4 SLFN11 expression is associated with favorable prognosis in immunotherapy-treated patients

**Figure 4:**
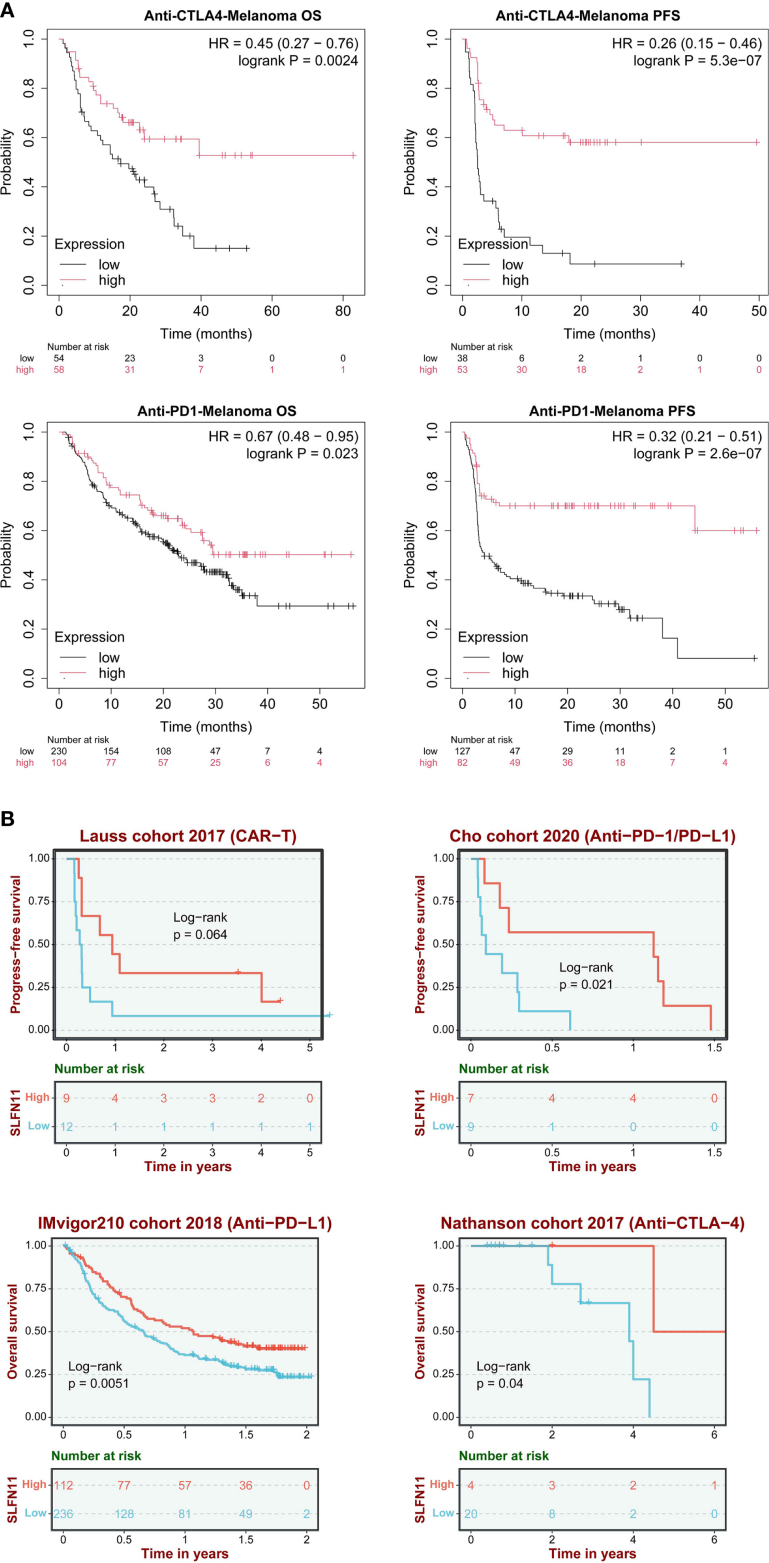
SLFN11 predicts immunotherapy response. **(A)** Hazard ratios (HRs) for OS and PFS in anti-CTLA-4 and anti-PD-1/PD-L1 cohorts (KMplot). **(B)** Validation in independent cohorts (Lauss 2017 CAR-T, IMvigor210, Cho cohort 2020 anti-PD1/anti-PDL1, Nathanson cohort 2017 anti-CTLA-4).

In anti-CTLA-4 treated cohorts, patients with high SLFN11 expression demonstrated significantly prolonged overall survival (OS; HR = 0.45, 95% CI 0.27–0.76, log-rank P = 0.0024) and progression-free survival (PFS; HR = 0.26, 95% CI 0.15–0.46, P = 5.3×10^-7^). Similarly, in anti-PD-1 therapy cohorts, high SLFN11 expression correlated with survival benefits (OS: HR = 0.67, 95% CI 0.48–0.95, P = 0.023; PFS: HR = 0.32, 95% CI 0.21–0.51, P = 2.6×10^-7^) (**Figure 4A**).

These findings were further validated across multiple independent cohorts: the Lauss 2017 CAR-T therapy cohort showed a trend toward improved PFS (P = 0.064), while the Cho 2020 (anti-PD-1/PD-L1, PFS, P = 0.021), IMvigor210 2018 (anti-PD-L1, OS, P = 0.0051), and Nathanson 2017 (anti-CTLA-4, OS, P = 0.04) cohorts consistently confirmed that high SLFN11 expression is associated with favorable prognosis in immunotherapy settings (**Figure 4B**). The consistent survival advantage observed across different immunotherapeutic modalities (immune checkpoint inhibitors and cellular therapy) and multiple validation cohorts supports SLFN11 as a potential prognostic indicator for melanoma patients undergoing immunotherapy. The association between SLFN11 and improved immunotherapy outcomes, together with our earlier observation that SLFN11 correlates with pro-inflammatory immune cell infiltration (**Figure 3**), raises the possibility that SLFN11 may impact the tumor immune microenvironment through immunomodulatory effects. To better understand how SLFN11 contributes to its prognostic role in tumor immune microenvironment, we next performed pathway enrichment analysis using the BEST database to identify potential immune-related molecular cascades linked to SLFN11, then conducted *in vitro* experiments to validate its effects on interactions between immune cells and tumor cells.

### 3.5 SLFN11 overexpression in melanoma cells may promote macrophage polarization toward an M1 phenotype and enhance CD8+ T cell cytotoxic activity in melanoma

Integrated analysis of the BEST database (Broad Institute’s Cancer Cell Line Encyclopedia and Tumor Gene Expression Analysis), Gene Set Enrichment Analysis (GSEA) indicated that higher SLFN11 expression was associated with enrichment of several immune-related pathways, including interferon gamma response (NES = 2.390; FDR = 4.9×10^-10^), interferon alpha response (NES = 2.304; FDR = 4.9×10^-10^), IL6-JAK-STAT3 signaling (NES = 1.98; FDR = 0.002) and allograft rejection (NES = 2.12; FDR = 1.1×10^-5^ (**Supplementary Figure 1**). These pathways are closely tied to antitumor immune processes in tumor. The interferon gamma/alpha response pathways drive CD8^+^ T cell activation and cytotoxic function (19); the IL-6/JAK/STAT3 signaling pathway potently activates inflammatory response via tumor-infiltrating immune cells in the tumor immune microenvironment (20), and the allograft rejection pathway reinforces immune recognition of tumor cells, via mechanisms shared with “foreign cell recognition” that enhance T cell targeting of tumor cells (21). These pathway enrichments raise the possibility that SLFN11 expression correlates with melanoma immunity. To investigate how SLFN11 may affect immune-related biological processes in a controlled experimental setting, we next performed *in vitro* studies using melanoma cell models, starting with the establishment of SLFN11-overexpressing cell lines to assess its impact on cell growth and immune cell interactions.

**Figure 5:**
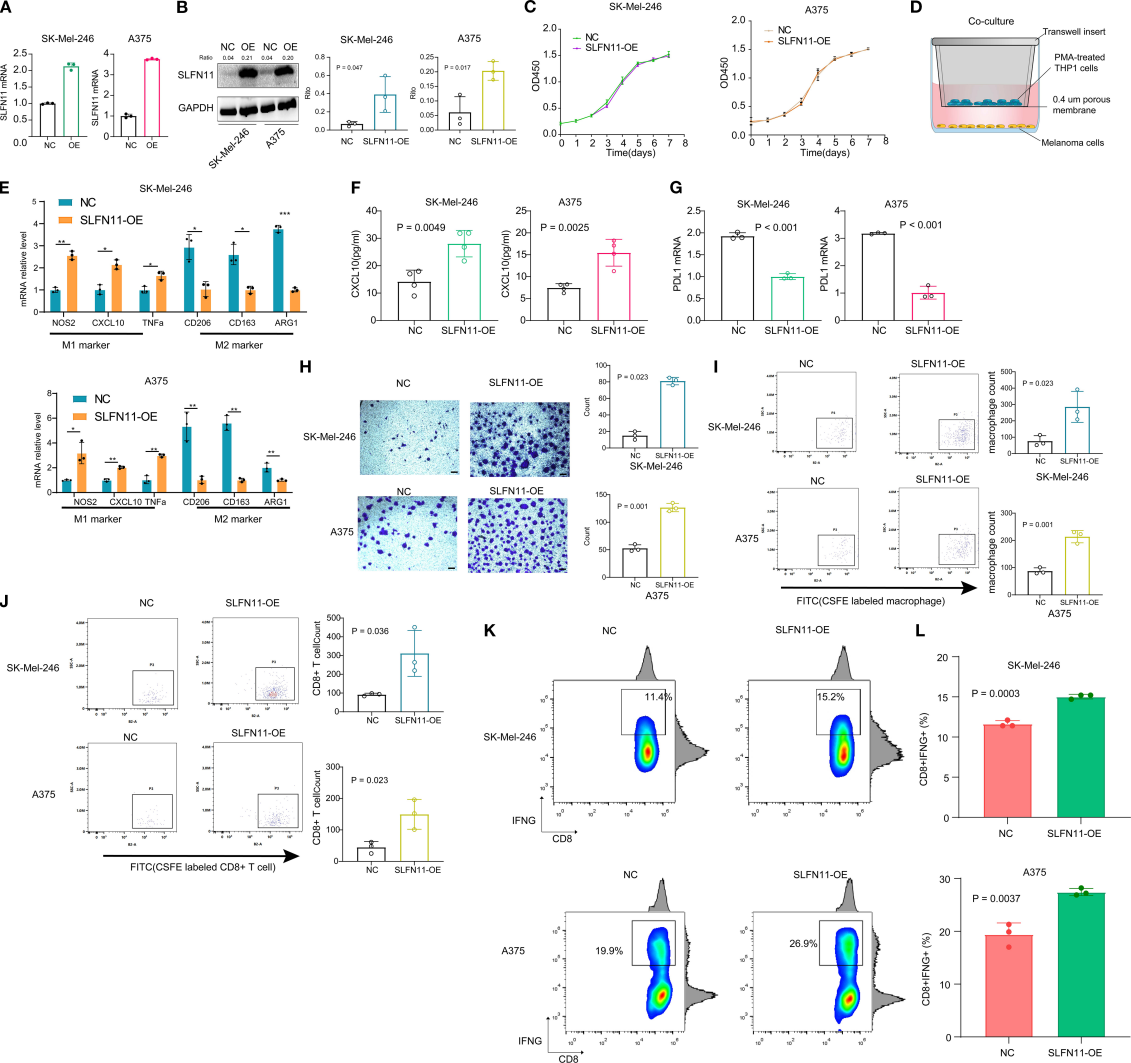
SLFN11 Overexpression Reprograms the Tumor Immune Microenvironment in Melanoma. (a, b) Validation of SLFN11-overexpressing (SLFN11-OE) and negative control (NC) SK-Mel-246 stable cell lines by PCR **(A)** and Western blot **(B)**. GAPDH served as loading control. **(C)** CCK-8 proliferation assay showing no significant difference in viability between SLFN11-OE and NC cells over 7 days (n = 3). **(D)** Schematic of co-culture system: SLFN11-OE or NC melanoma cells were co-cultured with PMA-induced THP-1 M0 macrophages. **(E)** qPCR analysis of macrophage polarization markers after co-culture. Macrophages exposed to SLFN11-OE cells exhibited upregulated M1 markers (NOS2, CXCL10, TNFα) and downregulated M2 markers (CD163, CD206, ARG1) compared to NC (n = 3, *p < 0.05, **p < 0.01, ***p < 0.001, two-tailed t-test). **(F)** ELISA quantification of CXCL10 in supernatants from macrophages isolated post-co-culture and maintained for 48 hours (n = 4). **(G)** PD-L1 mRNA levels in SLFN11-OE versus NC cells (n = 3). **(H)** Representative crystal violet staining images showing migration of CFSE-labeled THP-1-derived M0 macrophages to the lower surface of the upper transwell chamber after 48 hours of co-culture with SLFN11-overexpressing (SLFN11-OE) or control tumor cells (SK-Mel-246 and A375) in the lower chamber. Quantification indicates significantly more migrated macrophages in the SLFN11-OE group. **(I)** Flow cytometry analysis of CFSE^+^ macrophages in the lower chamber, confirming a higher proportion of migrated macrophages in the SLFN11-OE group compared to controls, consistent with enhanced chemotaxis. **(J)** Flow cytometry analysis of CFSE-labeled CD8^+^ T cells migrating to the lower transwell chamber after co-culture with SLFN11-OE or control tumor cells. **(K)** Percentage of CD8^+^IFNG^+^ cells after co-culture with SLFN11-overexpressing vs. control (NC) melanoma cells. **(L)** Quantification of CD8+IFNG+ T cell frequency, presented as box and bar plots. Error bars represent the mean ± SEM (n = 3). Statistical significance was determined using an unpaired Student’s t-test.

We successfully established stable SLFN11-overexpressing (SLFN11-OE) melanoma cell lines (SK-Mel-246 and A375) as well as negative control (NC) cell lines transfected with empty vectors. The successful integration of the plasmid and overexpression of SLFN11 were verified using polymerase chain reaction (PCR) (**Figure 5A**) and Western blot (WB) analysis (**Figure 5B**, **Supplementary Figure 2**). To evaluate the direct effect of SLFN11 on the growth of melanoma cells, we conducted CCK-8 proliferation assays. No significant difference in cell proliferation was detected between SLFN11-OE and NC cells (**Figure 5C**), suggesting that SLFN11 overexpression does not inherently influence the proliferative capacity of melanoma cells.

To explore possible immunomodulatory effects of SLFN11, SLFN11-overexpressing (SLFN11-OE) or negative control (NC) melanoma cells were co-cultured with THP-1-derived M0 macrophages (induced by PMA) (**Figure 5D**). Prior to these co-culture experiments, we validated the efficiency of macrophage polarization to ensure model reliability: THP-1 monocytes were treated with PMA to induce differentiation into M0 macrophages, followed by stimulation with LPS + IFNγ (to induce M1 polarization) or IL4 + IL13 (to induce M2 polarization) for 48 hours. qPCR analysis confirmed successful polarization: M1 macrophages showed significantly upregulated expression of M1 markers (NOS2, CXCL10, and TNFα) compared to M0 macrophages, while M2 macrophages exhibited marked upregulation of M2 markers (CD163, ARG1, and CD206). PMA-induced M0 macrophages showed no significant differences in M1 or M2 marker expression relative to untreated THP-1 monocytes, confirming their unpolarized phenotype (**Supplementary Figure 3**).

Furthermore, qPCR analysis revealed that macrophages co-cultured with SLFN11-OE cells displayed significant upregulation of M1 markers (NOS2, CXCL10, and TNFα) compared to those co-cultured with NC cells (**Figure 5E**). Conversely, M2 markers (CD163, CD206, and ARG1) were downregulated (**Figure 5E**). To further assess these trends, macrophages were isolated after co-culture and maintained in fresh medium for 48 hours; ELISA analysis showed a measurable increase in CXCL10 secretion in the supernatant of macrophages previously exposed to SLFN11-OE cells (**Figure 5F**). Additionally, SLFN11 overexpression was associated with downregulation of PD-L1 mRNA levels in both SK-Mel-246 and A375 cells (**Figure 5G**).

In transwell chemotaxis assays, both the SLFN11-overexpressing (SLFN11-OE) group and control (NC) group were initialized with the same number of CFSE-labeled M0 macrophages or CD8+ T cell in the upper chamber, and the lower chambers of both groups were seeded with an equal number of SLFN11-OE or NC melanoma cells, respectively. After 48 hours, crystal violet staining of cells migrating to the lower surface of the upper chamber indicated a small increase in macrophage recruitment by SLFN11-OE tumor cells (**Figure 5H**). Complementary flow cytometry analysis further confirmed a higher proportion of CFSE^+^ macrophages in the lower chamber of the SLFN11-OE group (**Figure 5I**). Similarly, in Transwell co-cultures assessing CD8^+^ T cell recruitment, flow cytometry quantification of migrating CFSE-labeled CD8^+^ T cells showed that SLFN11-OE tumor cells recruited a greater number of these cytotoxic T cells compared to NC tumor cells (**Figure 5J**). Together, these results demonstrate that SLFN11 overexpression in melanoma cells modestly enhances the recruitment two immune cell types of M0 macrophages and CD8^+^ T cells.

To evaluate potential functional consequences for antitumor immunity, a co-culture system was established using SLFN11-OE or NC melanoma cells (SK-Mel-246 and A375) and primary human CD8^+^ T cells isolated from healthy donor peripheral blood. Compared to NC-transfected tumor cells, co-culture with SLFN11-overexpressing cells was associated with a slight increase in the proportion of cytotoxic CD8^+^ T cells, as evidenced by elevated CD8^+^IFNG^+^ populations (A375: OE 27.43% ± 0.3930% vs NC 19.40% ± 1.266%; SK-Mel-246: OE 15.03% ± 0.1667% vs NC 11.63% ± 0.2333%) (**Figures 5K, L**). These observations suggest that tumor-intrinsic SLFN11 expression may be linked to subtle enhancements in CD8^+^ T cell effector functions, including increased production of the pro-inflammatory cytokine IFN-γ, which could modestly contribute to tumor cell killing capacity.

## 6 Discussion

The present study systematically characterizes the role of SLFN11 in melanoma, integrating multi-omics analyses and functional validation to reveal its expression patterns, prognostic significance, and immunomodulatory functions. Our findings provide preliminary insights into SLFN11’s association with melanoma prognosis and immune regulation, supporting its potential as a candidate biomarker and therapeutic target worthy of further study.

Our analysis revealed distinct expression patterns of SLFN11 in melanoma: mRNA levels are reduced in primary skin cutaneous melanoma (SKCM) compared to normal skin, yet significantly elevated in metastatic lesions relative to primary tumors. Primary tumors may downregulate SLFN11 to evade early immune surveillance, while metastatic lesions upregulate it as an adaptive response to the hostile microenvironment of distant organs. Distant metastatic lesions endure persistent metabolic stress, compelling cancer cells in these new niches to develop adaptive mechanisms to survive in otherwise unfavorable conditions (22, 23), and SLFN11 upregulation likely contributes to this adaptive process.

The consistent association between high SLFN11 expression and favorable survival outcomes in melanoma was validated across five independent cohorts for overall survival (OS) and two cohorts for progression-free survival (PFS), with borderline significance in additional datasets. Subgroup analyses further confirmed that this prognostic value persists across tumor stages (T1-T3), nodal statuses (N1-N4), and metastatic states (M0-M1), underscoring its reliability across diverse clinical scenarios. Multivariable Cox regression identified SLFN11 as an independent prognostic factor, unaffected by confounding variables such as age, gender, or pathologic stage. This observation aligns with emerging evidence that Schlafen family members exert context-dependent roles in tumor progression, with their functional outputs potentially modulated by microenvironmental cues like inflammatory status or therapy exposure (24–26). For instance, in glioblastoma, SLFN11 has been shown to regulate non-canonical NFκB signaling to promote tumor progression (27), whereas its association with survival benefits in melanoma stands in striking contrast to such oncogenic roles in other malignancies. This divergence suggests that tissue-specific molecular interactions may alter SLFN11’s functional role, with its association with favorable outcomes in melanoma contrasting with oncogenic roles in other cancers, though the underlying mechanisms require further clarification.

Our immunoinfiltration analysis reveals that SLFN11 exhibits strong co-expression with multiple cytotoxic lymphocytes (CD8^+^ T cells, natural killer cells) and key immune checkpoint molecules, including PD-L1, CTLA-4, and TIM-3, in melanoma. This finding aligns with recent reports linking Schlafen proteins to immune cell exhaustion pathways (9, 28), though the precise molecular mechanisms bridging SLFN11 and checkpoint regulation in melanoma remain to be clarified.

In macrophage polarization assays, co-culturing SLFN11-overexpressing melanoma cells with THP-1-derived M0 macrophages induced a shift toward an M1 phenotype, characterized by upregulated M1 markers (NOS2, CXCL10, TNFα) and downregulated M2 markers (CD163, ARG1, CD206). This polarization was functionally validated by increased CXCL10 secretion in macrophage supernatants, a chemokine critical for recruiting cytotoxic immune cells like CD8^+^ T cells and natural killer cells (29, 30). Given that M1 macrophages are key drivers of pro-inflammatory responses and antigen presentation (31), this shift is associated with a pro-inflammatory phenotype that may contribute to antitumor immunity, consistent with our observations that SLFN11 expression correlates with increased infiltration of M1 macrophages in melanoma.

We observed that overexpression of SLFN11 in melanoma cells leads to downregulated PD-L1 mRNA levels. Previous research has demonstrated that SLFN11 knockdown in hepatocellular carcinoma (HCC)cells promotes upregulates the expression of PD-L1 via activation of the NF-κB pathway (32). This suggests a potential conserved regulatory axis where SLFN11 negatively modulates PD-L1. Our observation of SLFN11 overexpression-associated PD-L1 downregulation in melanoma likely reflects the reversal of this pathway. SLFN11 may indirectly reduce PD-L1 expression by suppressing NF-κB and other inflammation-related signals. This regulatory pattern further links SLFN11 function to the immunosuppressive state within the tumor microenvironment, providing an additional mechanistic clue to how SLFN11 influences the efficacy of immune checkpoint inhibitors.

Concurrently, SLFN11 overexpression augmented CD8^+^ T cell-mediated responses: transwell assays showed increased recruitment of CFSE-labeled CD8^+^ T cells to SLFN11-overexpressing melanoma cells, while co-culture experiments revealed a rise in CD8^+^IFNG^+^ populations. This two-pronged effect, promoting M1 polarization to amplify inflammatory signaling, and boosting CD8^+^ T cell recruitment and IFN-γ secretion, creates a feed-forward loop that strengthens antitumor immunity. While prior studies have primarily focused on SLFN11’s role in DNA damage response and chemosensitivity (33, 34), we provide the clue that tumor-intrinsic SLFN11 overexpression functionally may remodel antitumor immunity by augmenting T cell effector functions.

There are some limitations. First, due to the focused scope on melanoma, despite systematic searches across public databases including TCGA, we were unable to obtain paired SLFN11 protein expression data from normal skin tissues and melanoma samples. This has restricted our ability to comprehensively compare SLFN11 expression differences between normal tissues and melanoma at the protein level, limiting related analyses to the mRNA level. Second, while our study observed associations between SLFN11 expression and PD-L1 levels, as well as macrophage polarization phenotypes, and preliminarily validated its impact on immune cell recruitment and activity through functional assays, the underlying molecular mechanisms remain underexplored. For instance, the specific signaling pathways through which SLFN11 regulates PD-L1 expression and the molecular details of how it mediates M1 macrophage polarization require further investigation. Additionally, tumor cell SLFN11 overexpression was found to enhance CD8^+^ T cell cytotoxicity, but this effect was less pronounced in the SK-Mel-246 cell line, suggesting potential cell line-specific variability. Therefore, whether SLFN11 in tumor cells exerts a regulatory effect on CD8^+^ T cell function requires validation in multiple additional melanoma cell lines to confirm its generalizability.

In conclusion, our multi-omics and functional analyses suggest that SLFN11 correlates with favorable prognosis in melanoma and is associated with remodeling of the immune microenvironment, including effects on macrophage polarization and T cell activity. These observations support the potential of SLFN11 as a candidate prognostic biomarker and provide a foundation for further studies to explore its suitability as a therapeutic target in melanoma.

## Data Availability

The original contributions presented in the study are included in the article/**Supplementary Material**. Further inquiries can be directed to the corresponding authors.

## Supplementary material

Supplementary Figure 1 GSEA results of immune-related pathways associated with SLFN11 high expression. Gene Set Enrichment Analysis (GSEA) plots of immune-related pathways significantly enriched in SLFN11-high melanomas from the BEST database.

Supplementary Figure 2 Original full membrane images of Western blot analysis for SLFN11 and GAPDH. Original full membrane images of Western blot (WB) analysis verifying SLFN11 overexpression in stable SLFN11-overexpressing (SLFN11-OE) and negative control (NC) melanoma cell lines (SK-Mel-246 and A375).

Supplementary Figure 3 Validation of macrophage polarization efficiency using qPCR analysis of M1/M2 marker genes. qPCR quantification of polarization marker genes in THP-1-derived macrophages to confirm the reliability of M0, M1, and M2 polarization models.
