# Supplementary material for: SLFN11 expression correlates with immune microenvironment and predicts prognosis in melanoma
Source: Front Immunol. 2025 Sep 22;16:1607056. doi: 10.3389/fimmu.2025.1607056 (PMC12497827; doi:10.3389/fimmu.2025.1607056)
Supplement: Supplementary Figure 1 — GSEA results of immune-related pathways associated with SLFN11 high expression. Gene Set Enrichment Analysis (GSEA) plots of immune-related pathways significantly enriched in SLFN11-high melanomas from the BEST database. [file DataSheet1.zip › Supplementary Figure 1.PDF]

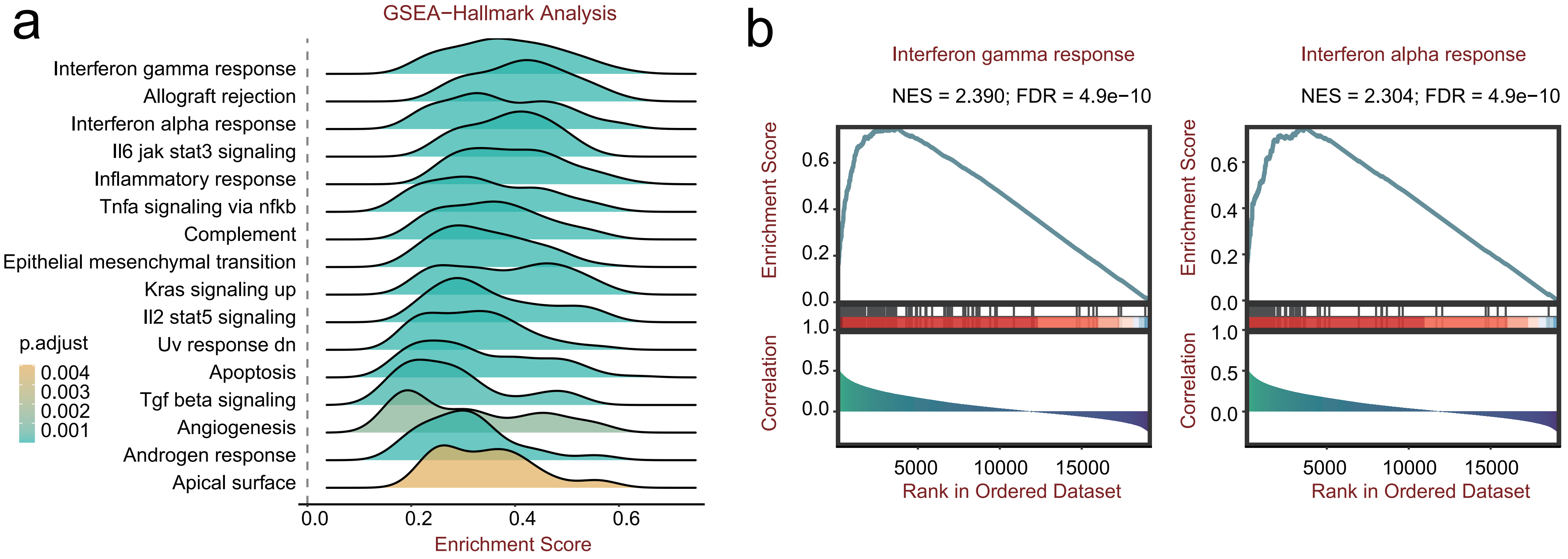
a
GSEA−Hallmark Analysis
Interferon gamma response
Allograft rejection
Interferon alpha response
Il6 jak stat3 signaling
Inflammatory response
Tnfa signaling via nfkb
Complement
Epithelial mesenchymal transition
Kras signaling up
Il2 stat5 signaling
Uv response dn
Apoptosis
Tgf beta signaling
Angiogenesis
Androgen response
Apical surface
p.adjust
0.004
0.003
0.002
0.001
0.0
0.2
0.4
0.6
Enrichment Score
b
Interferon gamma response
NES = 2.390; FDR = 4.9e−10
Interferon alpha response
NES = 2.304; FDR = 4.9e−10
Enrichment Score
Correlation
Rank in Ordered Dataset
5000
10000
15000
