# Supplementary figures and images for: SLFN11 expression correlates with immune microenvironment and predicts prognosis in melanoma
Source: Front Immunol. 2025 Sep 22;16:1607056. doi: 10.3389/fimmu.2025.1607056 (PMC12497827; doi:10.3389/fimmu.2025.1607056)

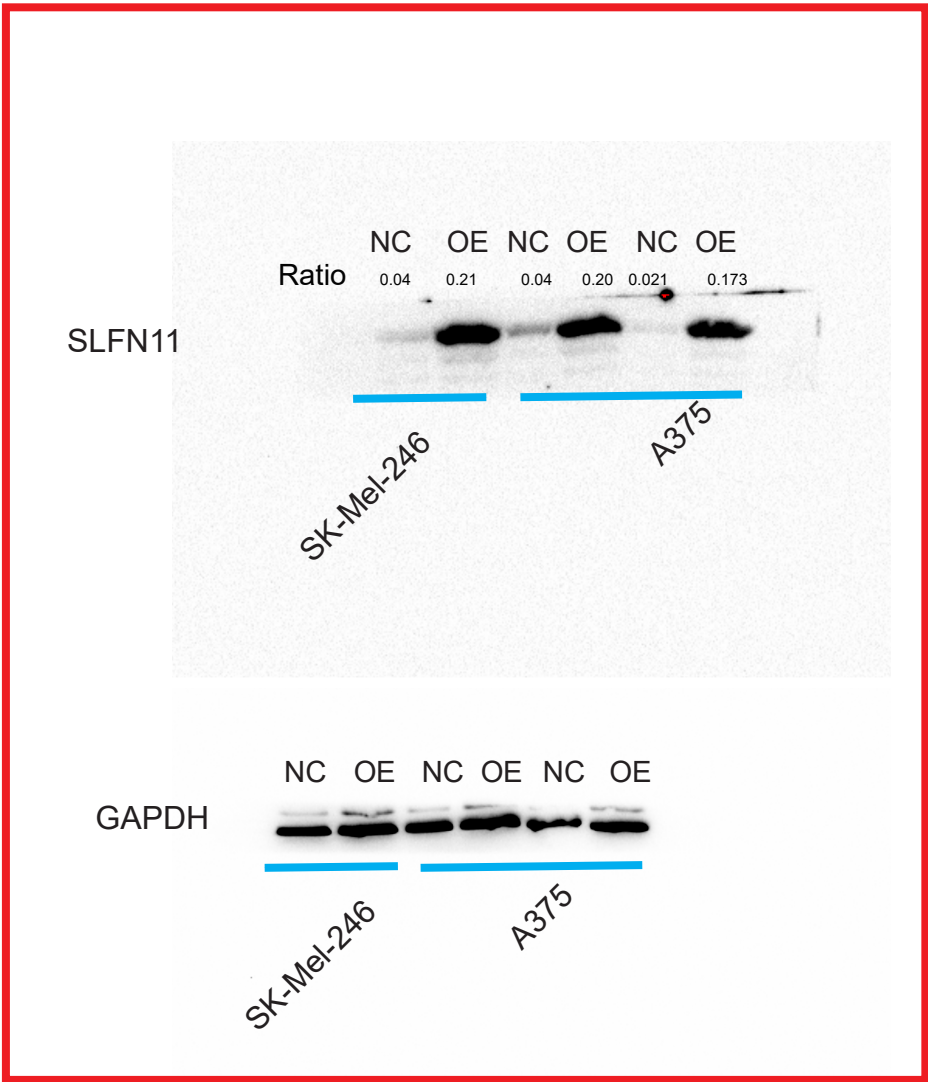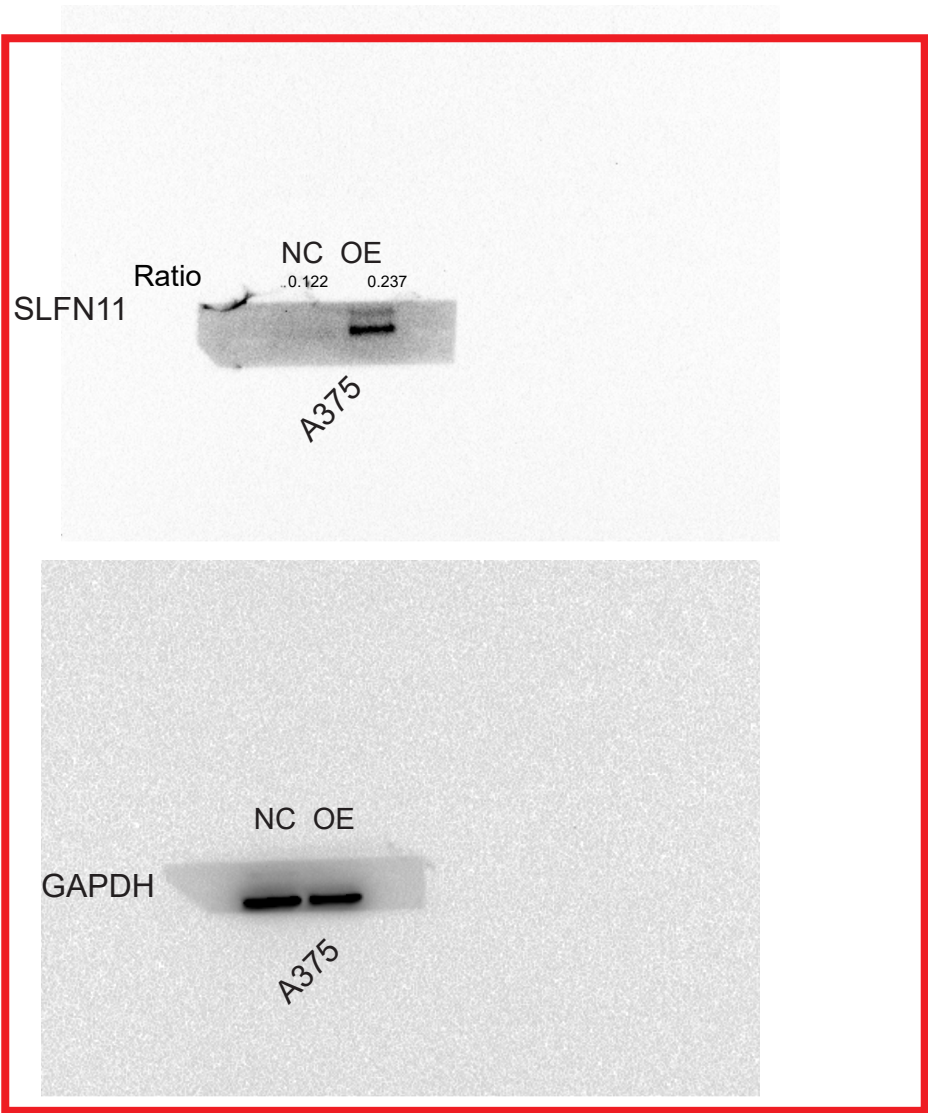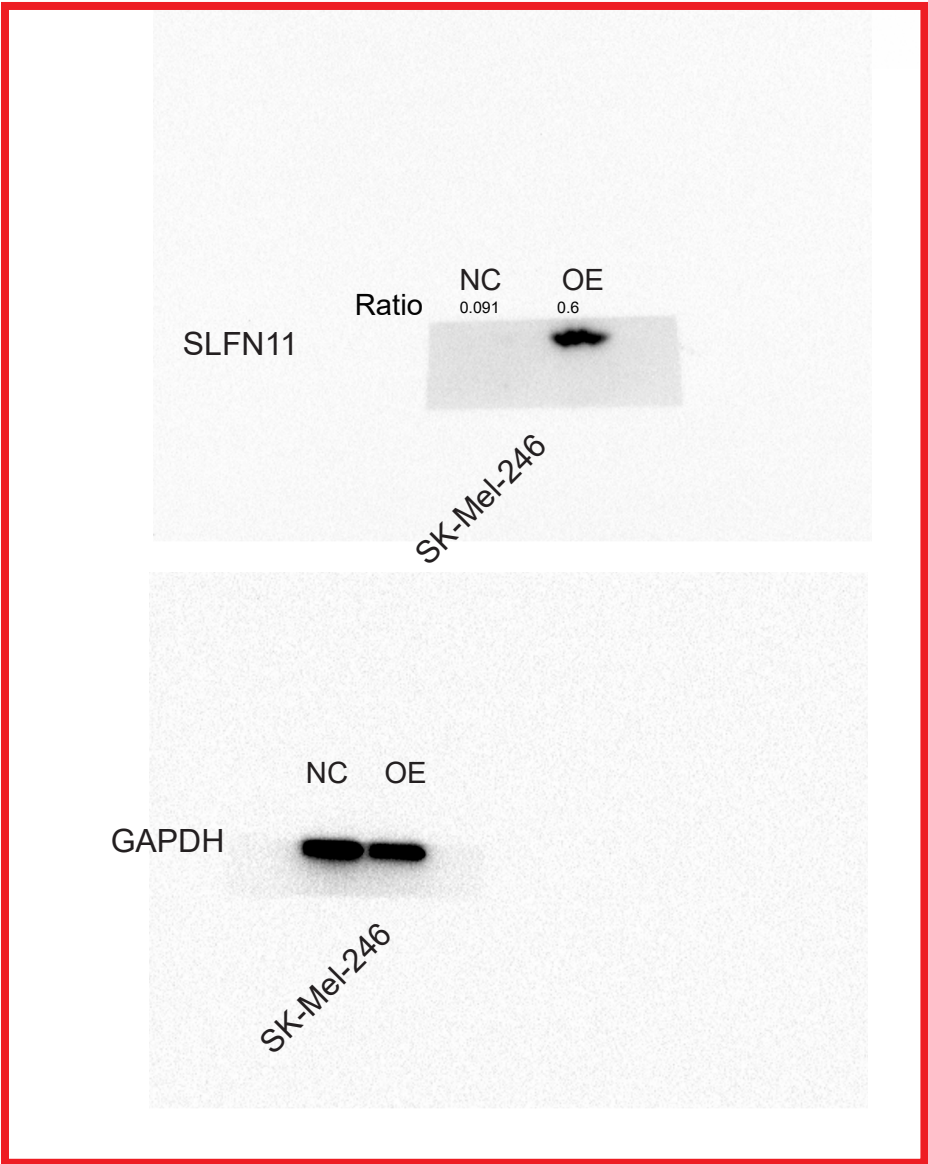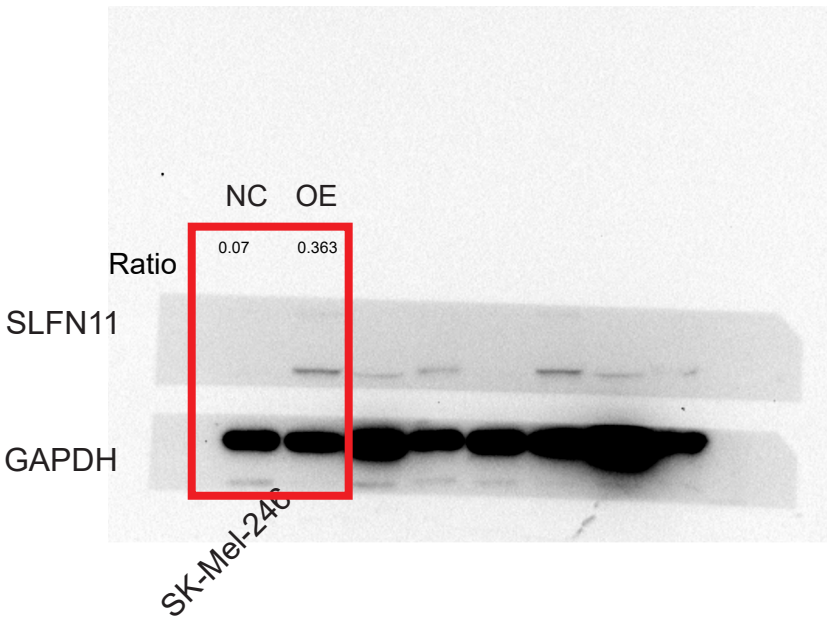

Supplement: Supplementary Figure 1 — GSEA results of immune-related pathways associated with SLFN11 high expression. Gene Set Enrichment Analysis (GSEA) plots of immune-related pathways significantly enriched in SLFN11-high melanomas from the BEST database. [file DataSheet1.zip › Supplementary Figure 3.PDF]

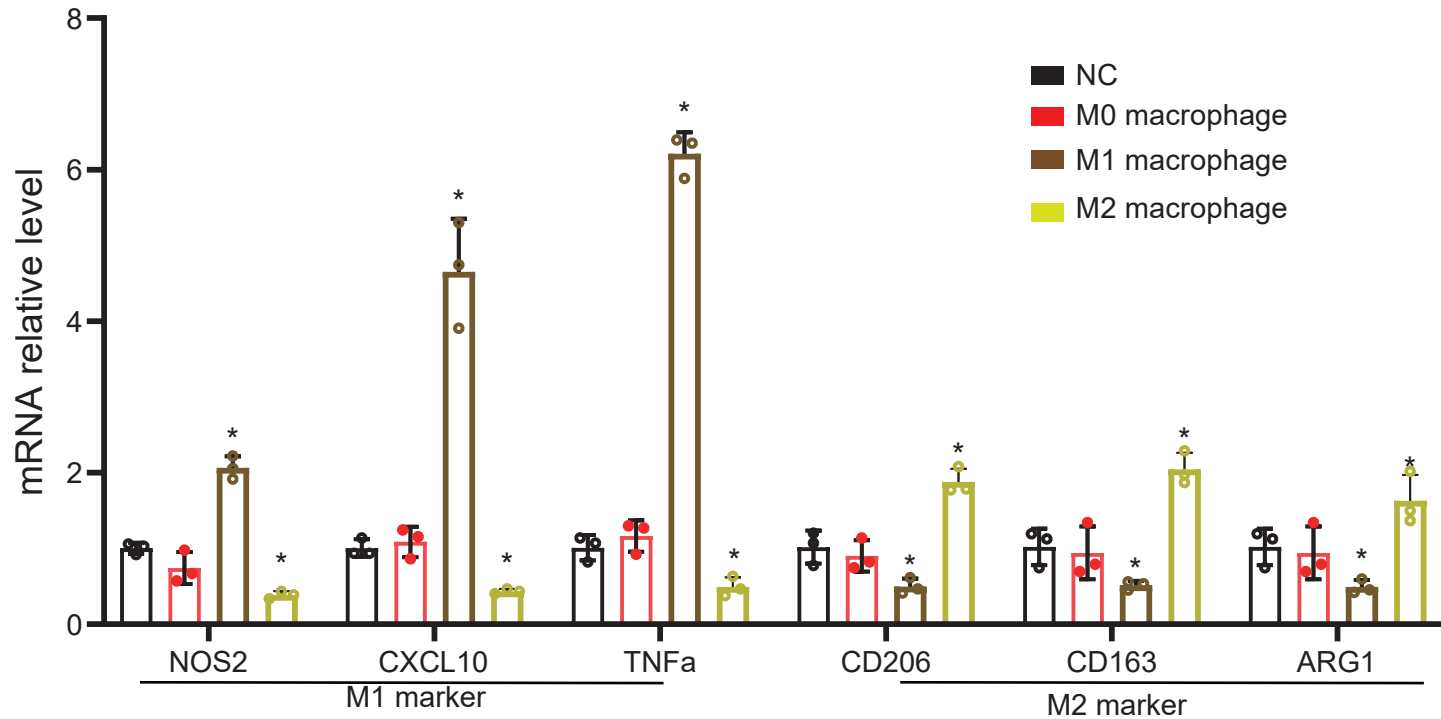

Supplement: Supplementary Figure 1 — GSEA results of immune-related pathways associated with SLFN11 high expression. Gene Set Enrichment Analysis (GSEA) plots of immune-related pathways significantly enriched in SLFN11-high melanomas from the BEST database. [file DataSheet1.zip › Supplementary Figure 2.PDF]
