# Supplementary material for: SLFN11 expression correlates with immune microenvironment and predicts prognosis in melanoma
Source: Front Immunol. 2025 Sep 22;16:1607056. doi: 10.3389/fimmu.2025.1607056 (PMC12497827; doi:10.3389/fimmu.2025.1607056)
Supplement: Supplementary file 2 [file Table1.docx]

Supplemental Table. Primers and interference sequences used in this study

| Gene | Forward primer |
| --- | --- |
| Human-NOS2-Forward | TTCAGTATCACAACCTCAGCAAG |
| Human-NOS2-Reverse | TGGACCTGCAAGTTAAAATCCC |
| Human-TNF-Forward | TTGAGGGTTTGCTACAACATGGG |
| Human-TNF-Reverse | GCTGCACTTTGGAGTGATCG |
| Human-CXCL10-Forward | GTGGCATTCAAGGAGTACCTC |
| Human-CXCL10-Reverse | TGATGGCCTTCGATTCTGGATT |
| Human-ARG1-Forward | GGCTGGTCTGCTTGAGAAAC |
| Human-ARG1-Reverse | ATTGCCAAACTGTGGTCTCC |
| Human-CD163-Forward | CGGCTGCCTCCACCTCTAAGT |
| Human-CD163-Reverse | ATGAAGATGCTGGCGTGACA |
| Human-SLFN11-Forward | AACCCCAACGCCCGATAAC |
| Human-SLFN11-Reverse | TCATGCAAGCATAGCCATAGAG |
| Human-GAPDH-Forward | AAGCTCATTTCCTGGTATGACAACG |
| Human-GAPDH-Reverse | TCTTCCTCTTGTGCTCTTGCTGG |
| Human-PDL1-Forward | TGGCATTTGCTGAACGCATTT |
| Human-PDL1-Reverse | TGCAGCCAGGTCTAATTGTTTT |
